# Repression of *Igf1* expression by Ezh2 prevents basal cell differentiation in the developing lung

**DOI:** 10.1242/dev.122077

**Published:** 2015-04-15

**Authors:** Laura A. Galvis, Aliaksei Z. Holik, Kieran M. Short, Julie Pasquet, Aaron T. L. Lun, Marnie E. Blewitt, Ian M. Smyth, Matthew E. Ritchie, Marie-Liesse Asselin-Labat

**Affiliations:** 1 ACRF Stem Cells and Cancer Division, The Walter and Eliza Hall Institute of Medical Research, Parkville, Victoria 3052, Australia; 2 Department of Medical Biology, The University of Melbourne, Parkville, Victoria 3052, Australia; 3 Department of Biochemistry and Molecular Biology, Monash University, Melbourne, Victoria 3800, Australia; 4 Bioinformatics Division, The Walter and Eliza Hall Institute of Medical Research, Parkville, Victoria 3052, Australia; 5 Molecular Medicine Division, The Walter and Eliza Hall Institute of Medical Research, Parkville, Victoria 3052, Australia; 6 Department of Mathematics and Statistics, The University of Melbourne, Parkville, Victoria 3052, Australia

**Keywords:** Polycomb repressive complex 2, Ezh2, Lung development, Basal cells, IGF1, Mouse

## Abstract

Epigenetic mechanisms involved in the establishment of lung epithelial cell lineage identities during development are largely unknown. Here, we explored the role of the histone methyltransferase Ezh2 during lung lineage determination. Loss of Ezh2 in the lung epithelium leads to defective lung formation and perinatal mortality. We show that Ezh2 is crucial for airway lineage specification and alveolarization. Using optical projection tomography imaging, we found that branching morphogenesis is affected in Ezh2 conditional knockout mice and the remaining bronchioles are abnormal, lacking terminally differentiated secretory club cells. Remarkably, RNA-seq analysis revealed the upregulation of basal genes in Ezh2-deficient epithelium. Three-dimensional imaging for keratin 5 further showed the unexpected presence of a layer of basal cells from the proximal airways to the distal bronchioles in E16.5 embryos. ChIP-seq analysis indicated the presence of Ezh2-mediated repressive marks on the genomic loci of some but not all basal genes, suggesting an indirect mechanism of action of Ezh2. We found that loss of Ezh2 de-represses insulin-like growth factor 1 (*Igf1*) expression and that modulation of IGF1 signaling *ex vivo* in wild-type lungs could induce basal cell differentiation. Altogether, our work reveals an unexpected role for Ezh2 in controlling basal cell fate determination in the embryonic lung endoderm, mediated in part by repression of *Igf1* expression.

## Abstract (precis)

**SUMMARY**: The histone methyltransferase Ezh2 inhibits basal cell differentiation in the mouse lung by depositing repressive marks on the promoter region of basal cell genes and by repressing *Igf1* expression.

## INTRODUCTION

In the mouse embryonic lung, an epithelial hierarchy has been proposed where multi-lineage progenitor cells give rise to the mature lung epithelial cells (Alanis et al., 2014). Airway cells including club, ciliated, neuroendocrine and goblet cells derive from early Sox9-positive precursors that acquire Sox2 expression and lose Sox9 expression (Alanis et al., 2014). From E16.5, alveolar type 1 and type 2 cells arise from bipotent alveolar progenitor cells present at the tip of the epithelium that express Sox9 and markers of the two alveolar lineages (Alanis et al., 2014; Desai et al., 2014; Rawlins et al., 2009a; Treutlein et al., 2014). Studies to identify transcription factors and signaling pathways that drive branching morphogenesis and lineage specification have shown that lung morphogenesis is orchestrated by intrinsic epithelial signaling pathways as well as crosstalk between the epithelium and the mesenchyme (Hogan et al., 2014; Rock and Hogan, 2011). However, epigenetic mechanisms that control lung development, maintenance of cell fate and lineage specification remain largely uncharacterized.

Polycomb group (PcG) proteins are important epigenetic regulators that act in synergy during development to deposit repressive marks that maintain tissue-specific gene expression into adulthood (Boyer et al., 2006). The polycomb repressive complex (PRC) 2 mediates methylation of lysine 27 on histone 3 (H3K27) via the catalytically active SET-domain-containing proteins Ezh2 and Ezh1, whereas the other two core PRC2 members, Suz12 and Eed, are required for complex stability (Cao and Zhang, 2004). The vast majority of research on PRC2 has been on its capacity to trimethylate H3K27 (H3K27me3), which is associated with repression of transcription. Repressive H3K27me3 deposition serves as a docking site for the recruitment of PRC2 itself, and allows recruitment of PRC1 (Morey and Helin, 2010). Mono- or divalent methylation of H3K27 (H3K27me1 and H3K27me2) has recently been described to be a function of PRC2 and to correlate with active transcription and maintenance of cell-type-specific enhancers (Ferrari et al., 2014), indicating that PRC2 may control both activation and repression of transcription.

The PcG proteins are important for stem cell maintenance and for cell fate determination during embryonic development, and disruption of epigenetic control can result in carcinogenesis (Boyer et al., 2006; Sauvageau and Sauvageau, 2010; Schwartz and Pirrotta, 2013). Loss of the PRC2 components *Ezh2*, *Suz12* or *Eed* results in severe defects during gastrulation that are consistent with PRC2-regulating genes involved in lineage specification (Bracken and Helin, 2009). PcG complexes have been shown to target developmentally important genes, including Hox gene clusters required for tissue patterning (Boyer et al., 2006). Ezh2 also regulates proliferation through repression of the potent cell cycle inhibitors *Cdkn2a* and *Cdkn2b* in progenitor cells of specific tissues, including the epidermis, mammary gland, pancreas and muscle (Chen et al., 2009; Ezhkova et al., 2009; Juan et al., 2011; Pal et al., 2013). Ezh2 is involved in maintenance of tissue specificity by repressing the expression of unrelated tissue-specific genes (Juan et al., 2011; Pal et al., 2013) or maintaining multi-potent progenitor cells to control temporal expression of differentiation genes (Ezhkova et al., 2009; Juan et al., 2011).

We generated mice in which the catalytic domain of Ezh2 was conditionally deleted in the lung epithelium (*Shh-cre;Ezh2^fl/fl^*). Ablation of Ezh2 in the epithelium resulted in perinatal lethality with defective lung development and altered differentiation of multiple lung epithelial lineages. Strikingly, RNA-seq profiling of epithelial cells showed a marked increase in gene expression corresponding to basal cell gene signature after loss of Ezh2 in the epithelium. Three-dimensional optical projection tomography (OPT) imaging for keratin 5 confirmed the presence of a layer of basal cells surrounding the airways of Ezh2-depleted lung epithelium from E16.5, suggesting proximalization of the distal airways in the absence of Ezh2. ChIP-seq analysis revealed enrichment for H3K27me3 repressive marks on some basal genes in the control lung epithelium that were lost after deletion of Ezh2, but the genomic loci of other basal genes, such as *Krt5* or *Trp63* were not marked by H3K27me3 in control lungs, suggesting that factors activating basal cell-specific gene transcription may be activated in the absence of Ezh2. We observed that *Igf1* was strongly overexpressed in Ezh2-depleted lungs and that treatment of wild-type lungs with IGF1 induced basal cell differentiation *ex vivo*. Overall, our results demonstrate that repression of *Igf1* expression by Ezh2 contributes to the regulation of basal cell differentiation during embryonic lung lineage specification.

## RESULTS

### Ezh2 is required for lung development and survival at birth

**Fig. 1.**
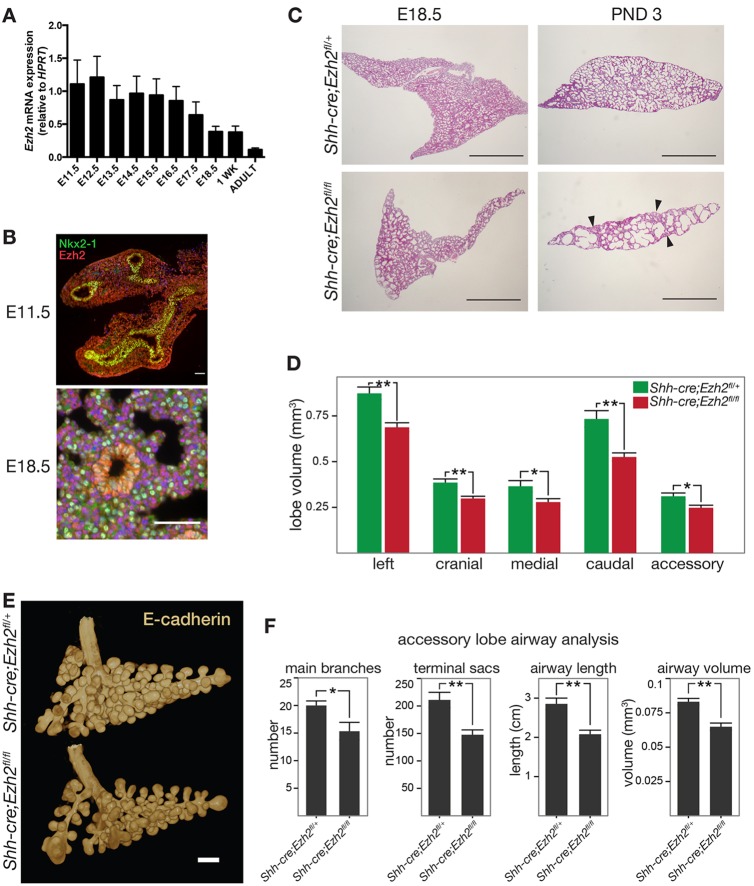
**Ezh2 is expressed throughout lung development and its deletion in the epithelium results in abnormal lung development.** (A) qRT-PCR analysis of *Ezh2* expression relative to *Hprt* from E11.5 to E18.5, in 1 week post-natal and in adult wild-type lung (*n*=4). Data represent mean±s.e.m. (B) Immunofluorescence staining of E11.5 and E18.5 lungs for Nkx2.1 (green) and Ezh2 (red). Scale bars: 50 µm. (C) Haematoxylin and Eosin staining of *Shh-cre;Ezh2^fl/fl^* and control lungs at E18.5 and post-natal day 3 (PND 3). Scale bars: 1 mm. Data are representative of *n*=10 or 11 animals. Arrowheads indicate region of atelectasis. (D) Whole lobe volume analysis of OPT-scanned *Shh-cre;Ezh2^fl/fl^* (*n*=6) and control (*n*=4) lungs at E14.5. Data represent mean±s.e.m. Unpaired two-tailed *t*-test, *P<0.05; **P<0.01. (E) Rendering of E-cadherin-stained accessory lobes of *Shh--cre;Ezh2^fl/fl^* and control lungs. Scale bar: 200 µm. (F) Accessory lobe branching in E-cadherin-stained OPT-imaged *Shh-cre;Ezh2^fl/fl^* (*n*=6) and control (*n*=4) lungs was quantified using Tree Surveyor software. Data represent mean±s.e.m. Unpaired two-tailed *t*-test with Welch's correction. *P<0.05; **P<0.01.

We first examined the expression of Ezh2 during embryonic lung morphogenesis, after birth and in the adult. Quantitative RT-PCR results showed high levels of *Ezh2* expression throughout development from E11.5 to E17.5 followed by a decrease at E18.5, reaching the lowest levels in adulthood (Fig. 1A). Confocal immunofluorescence for Ezh2 and Nkx2.1, a marker of lung epithelial cells, indicated that Ezh2 expression is predominantly nuclear and is detected in the mesenchyme and epithelium at E11.5 but becomes restricted to the airway epithelium from E18.5 (Fig. 1B; supplementary material Fig. S1A). To evaluate the role of Ezh2 in lung epithelium, we generated *Shh-cre;Ezh2^fl/fl^* mice in which *Ezh2* was efficiently excised from E9.5 in the epithelium of the lung primordia. As the *cre* allele was knocked into the *Shh* locus, resulting in loss of one *Shh* allele, *Shh-cre;Ezh2^fl/+^* animals were used as controls. PCR analysis of genomic DNA and cDNA from lung epithelial cells sorted based on the expression of EpCAM (McQualter et al., 2010) confirmed the excision of the SET domain of Ezh2 specifically in the epithelium of conditionally targeted mice (supplementary material Fig. S1B,C). *Shh-cre;Ezh2^fl/fl^* mice showed perinatal mortality with the majority of the pups dying within the first 2 days of birth. Only one animal survived to adulthood (supplementary material Table S1) and no gross lung defects were evident (data not shown). Genomic DNA analysis showed incomplete excision of the *Ezh2* floxed allele in this animal, explaining the absence of a phenotype (supplementary material Fig. S1D). Histological examination of *Shh-cre;Ezh2^fl/fl^* pups at birth revealed severe lung morphological abnormalities. The lungs had enlarged air sacs with areas of collapsed lung (atelectasis) and resembled an emphysema phenotype (Fig. 1C). To explore the phenotype of *Shh-cre;Ezh2^fl/fl^* lungs, we performed 3D imaging of E-cadherin stained E14.5 lungs using OPT. Ezh2 conditional knockout mice had smaller lungs compared with controls, as evaluated by measuring the whole lung volume (supplementary material Fig. S1E) and individual lobe volumes (Fig. 1D). Detailed analysis of the epithelial tree in the accessory lobe using Tree Surveyor software (Combes et al., 2014; Short et al., 2013) showed differences in the lung morphology of *Shh-cre;Ezh2^fl/fl^* lungs compared with controls. A significant reduction in the number of branches associated with a reduced number of terminal sacs was observed (Fig. 1E,F). The airways were shorter and their volume was reduced (Fig. 1F) but their diameters, curvature and angles did not differ significantly from those in controls (data not shown).

**Fig. 2.**
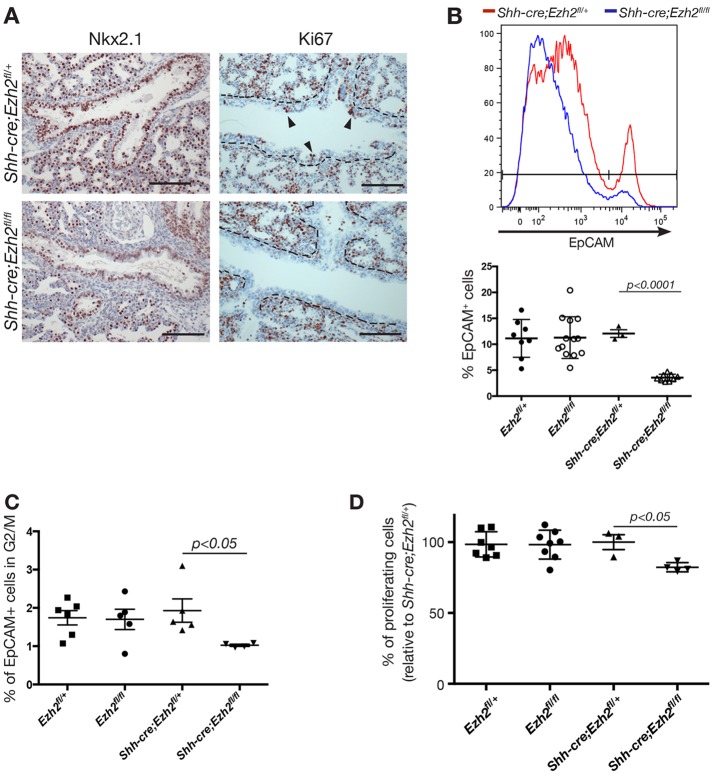
**Ezh2 deletion reduces epithelial cell proliferation in the embryonic lung.** (A) Immunohistochemistry showing Nkx2.1 and Ki67 in E18.5 lungs (*n*=10). Scale bars: 200 µm. Arrowheads indicate Ki-67-positive cells lining the airways. (B) Representative FACS histogram and percentages of CD31^−^CD45^−^EpCAM^+^ cells (mean±s.e.m.; unpaired *t*-test) in E18.5 Ezh2-deficient and control lungs. (C) Cell cycle analysis showing the percentage of cells in G2/M in control and *Shh-cre;Ezh2^fl/fl^* E16.5 lungs. Cells were CD31^−^CD45^−^EpCAM^+^. Data represent mean±s.e.m. *n*>4 animals per group; unpaired *t*-test. (D) *In vitro* proliferation assay of EpCAM^+^ cells isolated from E18.5 embryos shows reduced proliferation of *Shh-cre;Ezh2^fl/fl^* cells *in vitro*. *n*=3 independent experiments. Data represent mean±s.e.m. Unpaired *t*-test.

To further investigate the phenotype of Ezh2 conditional knockout mice, we evaluated epithelial cell numbers in E18.5 lungs. Immunostaining for Nkx2.1 showed a reduction in the number of Nkx2.1-expressing cells in *Shh-cre;Ezh2^f/lfl^* lungs compared with control animals (37±3.3% and 53±5.7% of all lung cells, respectively; Fig. 2A). The decrease in epithelial cells in *Shh-cre;Ezh2^f/lfl^* lungs was confirmed by analysis of EpCAM expression by flow cytometry (Fig. 2B) (3.56±0.2% and 12.1±0.7% EpCAM^+^ cells in *Shh-cre;Ezh2^f/lfl^* and control lungs, respectively; *P*=0.02, unpaired *t*-test). To determine whether the reduction in epithelial cellularity was due to increased apoptosis or to reduced proliferation, we analyzed cleaved caspase 3 and Ki67 expression. Although no changes in cleaved caspase 3 expression were observed between knockout and control mice (data not shown), loss of Ki67 expression was observed in *Shh-cre;Ezh2^fl/fl^* airway cells (Fig. 2A). Detailed analysis of cell cycle stages demonstrated that loss of Ezh2 affected progression through the cell cycle with a reduction in the percentage of epithelial cells (EpCAM^+^) in G2/M at E16.5 compared with control animals (Fig. 2C). These results were confirmed with an *in vitro* proliferation assay where a significantly reduced proliferative capacity of *Shh-cre;Ezh2^fl/fl^* EpCAM^+^ sorted cells compared with controls was observed (Fig. 2D). These data suggest that Ezh2 is essential for normal lung branching morphogenesis and controls proliferation of lung epithelial cells.

### Loss of Ezh2 results in perturbed airway lineage specification and a defect in alveoli formation

**Fig. 3.**
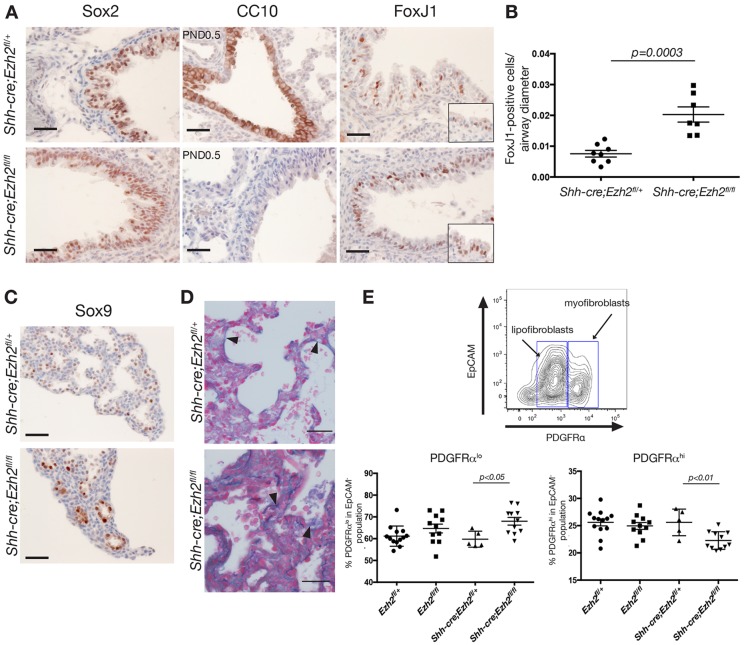
**Ezh2 deletion causes abnormal bronchiolar epithelium differentiation and perturbed alveolar formation.** (A) Immunohistochemical staining for an early bronchial epithelial marker (Sox2), a club cell marker (CC10) and a ciliated cell marker (Foxj1). Scale bars: 25 µm. Images are representative of 10 E18.5 animals for Foxj1 and Sox2, and 11 postnatal lungs for CC10. (B) Number of Foxj1-positive cells normalized to airway diameter in *Ezh2*-depleted and control lungs (*n*=7-10). Data represent mean±s.e.m. Unpaired *t*-test (C) Representative immunohistochemical staining for early alveolar marker Sox9 in E18.5 lungs (*n*=8 or 9). Scale bars: 25 µm. (D) Representative GAF (Gomori's aldehyde fuchin) staining of post-natal *Shh-cre;Ezh2^fl/fl^* and control lungs at E18.5 (*n*=9). Arrowheads indicate elastic tissue fibers (purple). Scale bars: 20 µm. (E) FACS analysis of PDGFRα expression in CD31^−^CD45^−^EpCAM^−^ cells. Data represent mean±s.e.m. *n*>4. Unpaired *t*-test.

The lung phenotype of *Shh-cre;Ezh2^fl/fl^* mice suggested a perturbation in epithelial cell differentiation. To assess whether Ezh2 controlled lineage specification, we first evaluated the airway cell lineage and analyzed the expression of Sox2, a marker of airway progenitor cells in E18.5 embryos. No significant changes were observed in Sox2 expression (Fig. 3A), suggesting that airway precursor cells form normally in the remaining airways of conditional knockout mice. However, expression of the club cell-specific marker CC10 was completely abolished in *Ezh2*-depleted airways (Fig. 3A). To further investigate whether Ezh2 loss inhibited secretory cell specification, we assessed expression of secretoglobin 3a2 (Scgb3a2), a marker of club cell precursors (Tsao et al., 2009). No change in the expression of Scgb3a2 was observed in Ezh2-deficient lungs (supplementary material Fig. S2A,B), suggesting that cells were specified towards the secretory lineage but could not reach full maturation. Interestingly, expression of the ciliated cell markers Foxj1 (Fig. 3A,B) and acetylated tubulin (supplementary material Fig. S2B) was increased in proximal airways, suggesting that the balance between ciliated and secretory cells was deregulated in the proximal airways of Ezh2-deficient lungs. The balance between ciliated cells and secretory cells during lung morphogenesis is controlled by Notch signaling (Tsao et al., 2009). However, downregulation of the Notch1 intracellular domain was not observed in *Ezh2*-deficient epithelial cells (data not shown), suggesting that Ezh2 is unlikely to regulate the Notch pathway during embryonic lung development. We then assessed whether other airway lineages were perturbed in *Ezh2*-deficient lungs and evaluated the presence of neuroendocrine cells by immunohistochemistry and mucin-producing goblet cells by periodic acid-Schiff staining. No discernible differences were observed for these two lineages between *Ezh2*-deficient and control lungs (data not shown). Given the absence of club cell specification in Ezh2-depleted lungs, we investigated whether specific deletion of Ezh2 in club cells would affect the lung phenotype of these animals. *Scgb1a1-cre^ERT2^* mice were crossed with *Ezh2^fl/fl^* mice and recombination induced by administration of tamoxifen in E17.5 dams. The pups survived at birth and the lungs were collected at 1 week. Histological examination did not reveal any gross abnormality, whereas immunostaining for CC10 and Ki67 did not show any alterations (supplementary material Fig. S2C). These results suggest that depletion of Ezh2 in specified secretory cells at a late stage of lung morphogenesis does not affect cell specification and proliferation. However, in the early phase of development, Ezh2 controls the formation of airways and is required for the full maturation of Sox2^+^ Scgb3a2^+^ airway progenitor cells into club cells.

We then examined the effect of Ezh2 loss on alveoli formation. In newborn animals, *Ezh2*-deficient lungs displayed enlarged air sacs with areas of atelectasis, suggestive of a defect in alveolar cell differentiation and/or alveolar septation (Fig. 1C). Sox9 is an early marker of alveolar progenitor cells whose expression is completely abolished by E18.5 when alveolar cells have matured (Okubo et al., 2005). However in *Ezh2*-deficient lungs we observed that Sox9 remained expressed in the distal lung at E18.5 (Fig. 3C), suggesting a failure or delay in the maturation of Sox9-positive precursor cells. Nevertheless, no difference in the expression of markers of alveolar type II (pro-SP-C) and type I (T1α) cells was observed between conditional knockout and control animals (supplementary material Fig. S2D). This led us to investigate whether loss of Ezh2 in the epithelium could affect signaling to the mesenchyme and alter septa formation. Alveolar septa formation is dependent on crosstalk between the epithelium, endothelium and mesenchyme. No alteration in the number of CD31^+^ endothelial cells in *Shh-cre;Ezh2^fl/fl^* lungs was apparent (data not shown). In the mesenchyme, alveolar myofibroblasts are crucial for alveolar septation. Myofibroblasts (PDGFRα^hi^) are thought to differentiate from lipofibroblasts (PDGFRα^lo^) and are responsible for elastin deposition at the tip of developing septa with elastin deposition being required for the formation and growth of functional alveoli (Shifren et al., 2007; Wendel et al., 2000). Analysis of elastin formation in *Shh-cre;Ezh2^fl/fl^* mice by Gomori's aldehyde fuchsin (GAF) staining showed that the elastin fibers in *Shh-cre;Ezh2^fl/fl^* alveoli appeared thicker, shorter and more curved than in age-matched control lungs (Fig. 3D). FACS analysis of PDGFRα in lung mesenchyme showed that the ratio between lipofibroblasts and myofibroblasts was significantly altered in the *Ezh2*-deleted lungs, with a lower percentage of myofibroblasts and a higher percentage of lipofibroblasts compared with control mice (Fig. 3E). This observation suggests that lipofibroblast differentiation into myofibroblasts is altered in *Ezh2*-deficient lungs. Given that deletion of *Ezh2* in our model is restricted to the epithelium, the changes observed in the myofibroblast/lipofibroblast ratio imply that *Ezh2* may control the expression of genes involved in epithelial-mesenchymal crosstalk.

### Loss of Ezh2 leads to dramatic upregulation of gene expression associated with loss of the H3K27 tri-methylation mark

**Fig. 4.**
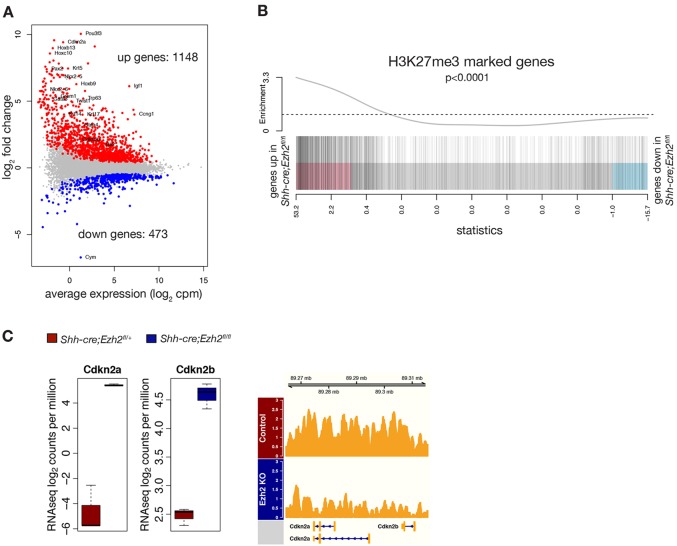
***Ezh2* loss induces dramatic changes in gene expression levels in the developing lung epithelium.** (A) MA plot showing differentially expressed genes (upregulated in red, downregulated in blue) between Ezh2-deficient and control epithelial cells in E16.5 embryonic lung. (B) ChIP-seq analysis of H3K27 tri-methylation revealed that H3K27me3-marked genes were predominantly upregulated in Ezh2-deficient epithelium (gene set test *P*<0.0001). Index marks represent the genes marked by H3K27me3 in control epithelium. (C) Box plot showing RNA-seq expression values [normalized log_2_ counts per million (cpm), *n*=3, FDR<0.001] for *Cdkn2a* and *Cdkn2b*, and a genome browser view of H3K27 tri-methylation over the *Cdkn2a* and *Cdkn2b* genomic locus.

To further examine the molecular mechanisms responsible for the abnormal phenotype of *Ezh2*-deficient lungs, we performed RNA-seq in sorted stromal (EpCAM^−^) and epithelial (EpCAM^+^) cell populations using *Shh-Cre;Ezh2^fl/fl^* and *Shh-Cre;Ezh2^fl/+^* lungs at E16.5. We observed substantial changes in gene expression in *Ezh2*-deficient epithelium with 1148 upregulated genes (FDR<0.05), consistent with the known repressive function of Ezh2 (Fig. 4A; supplementary material Table S2). ChIP-seq analysis of H3K27me3 modifications in control lung epithelium compared with Ezh2-deficient epithelium confirmed a correlation between loss of H3K27me3 marks and gene upregulation in *Shh-cre;Ezh2^fl/fl^* epithelium, suggesting that the effect of Ezh2 loss on gene expression is predominantly PRC2 dependent (Fig. 4B; supplementary material Table S3, gene set test, *P*<0.0001). Downregulated genes were also observed to a lesser extent (473 genes, FDR<0.05) but displayed lower fold changes compared with upregulated genes (Fig. 4A). The genes repressed in response to Ezh2 deletion may reflect an indirect effect of Ezh2 ablation or loss of transcriptionally active H3K27me1/2 marks in the absence of Ezh2 (Ferrari et al., 2014). Interestingly, a small number of differentially expressed genes were also observed in the stromal cells (11 genes, FDR<0.05, supplementary material Fig. S3A) where Ezh2 is not deleted, further suggesting that loss of *Ezh2* in the epithelium results in perturbed epithelial-mesenchymal interactions.

Ezh2 regulates cell proliferation in part by deposing H3K27me3 repressive marks on the *Ink4a/ARF* locus (*Cdkn2a, Cdkn2b*) (Bracken et al., 2007; Chen et al., 2009; Ezhkova et al., 2009; Juan et al., 2011; Pal et al., 2013). We found that the cell cycle inhibitors *Cdkn2a* and *Cdkn2b* were overexpressed in *Ezh2*-deficient epithelium (Fig. 4C, FDR<0.0001), most likely explaining the reduced proliferation observed at E18.5 in *Shh-cre;Ezh2^fl/fl^* animals and the reduced number of cells in G2/M (Fig. 2C). This increased expression coincided with the loss of H3K27me3 repressive marks at the *Cdkn2a/Cdkn2b* locus in the epithelium of *Shh-cre;Ezh2^fl/fl^* animals (Fig. 4C, FDR<0.01).

Given that PcG proteins have been implicated in the regulation of homeobox genes (Boyer et al., 2006), we investigated whether there were any changes in the Hox gene paralog groups 1 to 8 that are predominantly expressed in the lung (Mollard and Dziadek, 1997). Although Hox genes 1 to 8 were highly expressed in the lung mesenchyme and present at low levels in the epithelium of control lungs, this gene group was dramatically upregulated in Ezh2-deficient epithelium (FDR<0.01) but was not affected in the stroma (supplementary material Fig. S3B). Elevated expression was not limited to mesenchyme-specific Hox genes, but also to more posterior Hox genes (paralogs 9-13). This observation was in line with the loss of H3K27me3 marks across the entirety of each of the *Hox* loci in *Ezh2*-deficient epithelium (supplementary material Fig. S3C). To investigate whether deregulation of Hox genes was responsible for the phenotype observed in Ezh2-depleted lungs, E11.5 wild-type lungs were cultured *ex vivo* in the presence of retinoic acid, an inducer of Hox genes expression (Simeone et al., 1990). However, no gross morphological defects were observed in these lungs, suggesting that derepression of Hox genes is not solely responsible for the abnormal phenotype of *Shh-cre;Ezh2^fl/fl^* lungs (data not shown).

In murine ES cells, PcG proteins repress transcriptional regulators and genes involved in morphogenesis and organogenesis (Boyer et al., 2006), suggesting that PRC2 may repress non-related tissue-specific genes in a particular organ. To identify whether loss of Ezh2 in the lung epithelium resulted in upregulation of non-lung-specific genes, we derived tissue-specific gene expression signatures from 49 solid tissues using GNF Mouse GeneAtlas V3 data (GEO accession number, GSE10246). A substantial proportion (11 to 25%) of genes upregulated in Ezh2-deficient lungs overlapped with genes specifically expressed in non-lung tissues (supplementary material Fig. S3D), suggesting that Ezh2 is involved in regulating tissue-specific gene expression in the lung. These results show that in the lung endoderm, Ezh2 regulates tissue-specific genes involved in cell proliferation and tissue patterning.

### Ezh2 is required to repress basal gene expression in the lung epithelium

**Fig. 5.**
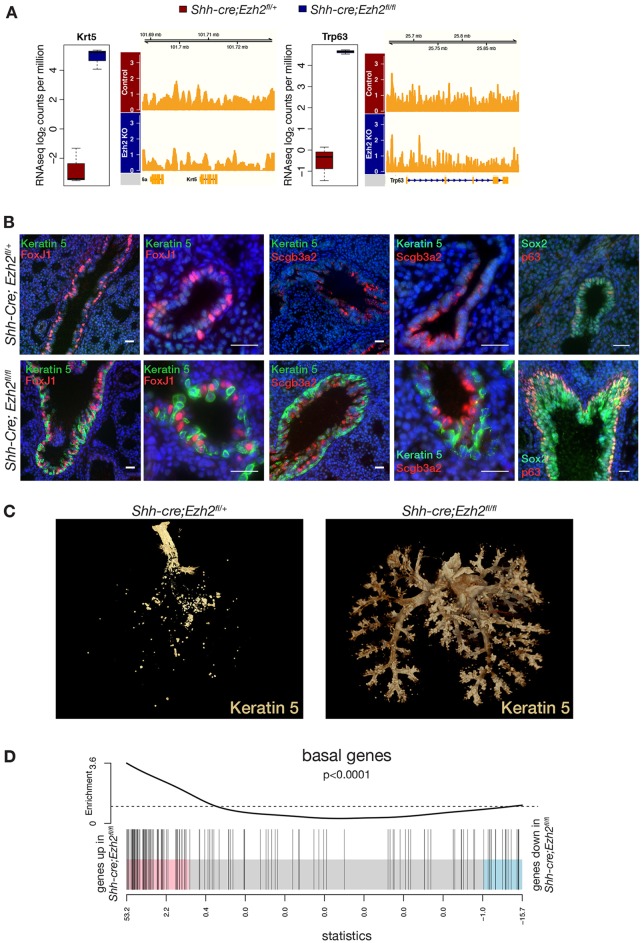
**Loss of *Ezh2* in the lung epithelium results in de-repression of basal gene expression.** (A) Box plot showing RNA-seq expression values (normalized log_2_cpm, *n*=3, FDR<0.01) for keratin 5 (*Krt5*) and *Trp63*, and a genome browser view of H3K27 tri-methylation over the corresponding loci. (B) Immunofluorescence staining for keratin 5, Scgb3a2, Foxj1, Sox2 and p63 in E17.5 control and *Shh-cre;Ezh2^fl/fl^* lungs. Scale bars: 25 µm. (C) OPT imaging of E16.5 control and *Shh-cre;Ezh2^fl/fl^* lungs stained with keratin 5. (D) Basal cell signature genes from adult trachea (Rock et al., 2009) were significantly enriched among the genes upregulated in Ezh2-ablated lung epithelium (gene set test *P*<0.0001). Index marks indicate genes from basal signature.

Most surprisingly, lung basal cell markers *Krt5*, *Krt14* and *Trp63* were among the top upregulated genes in *Ezh2*-deficient epithelium (Figs 4A, 5A, FDR<0.01). These data were confirmed by analysis of keratin 5 and p63 protein expression by immunostaining (Fig. 5B), demonstrating the presence of a layer of basal cells surrounding the proximal and distal airways only in *Shh-cre;Ezh2^fl/fl^* animals at E17.5. To determine the earliest time-point at which these basal cells appeared in Ezh2-depleted lung, keratin 5 immunohistochemistry was performed at E15.5, E16.5 and E17.5, and demonstrated the appearance of basal cells from E16.5 in *Shh-cre;Ezh2^fl/fl^* lungs (supplementary material Fig. S4A). OPT three-dimensional imaging of E16.5 lungs stained with keratin 5 further revealed the presence of keratin 5-positive cells throughout the branching network from proximal to distal airways in *Ezh2*-deficient embryonic lung, whereas keratin 5 staining in control lungs was largely confined to the trachea (Fig. 5C; see supplementary material Movies 1 and 2). Basal cells in *Shh-cre;Ezh2^fl/fl^* lungs did not co-express the markers of differentiated airway lineages Scgb3a2 and Foxj1 but expressed the airway precursor cell marker Sox2 (Fig. 5B), suggesting that basal cells may arise from Sox2-positive progenitor cells.

To further explore whether Ezh2 maintains lung epithelial lineage specification through repression of basal genes, we compared the expression of differentially expressed genes in *Ezh2*-ablated epithelium with the gene signature of adult mouse tracheal basal cells derived from Rock et al. (2009). Gene set testing revealed a strong enrichment for basal signature genes among genes overexpressed in the absence of *Ezh2* (Fig. 5D, *P*<0.0001). We observed enrichment for H3K27me3 marks around promoters of some lung basal genes in the control epithelium (supplementary material Fig. S4B, gene set test, *P*=0.0001) that disappeared after loss of Ezh2, demonstrating that Ezh2 plays a key role in repressing basal gene expression in the lung epithelium to maintain proximal to distal differentiation.

### Increased expression of *Igf1* in Ezh2-deficient lung contributes to basal cell differentiation

**Fig. 6.**
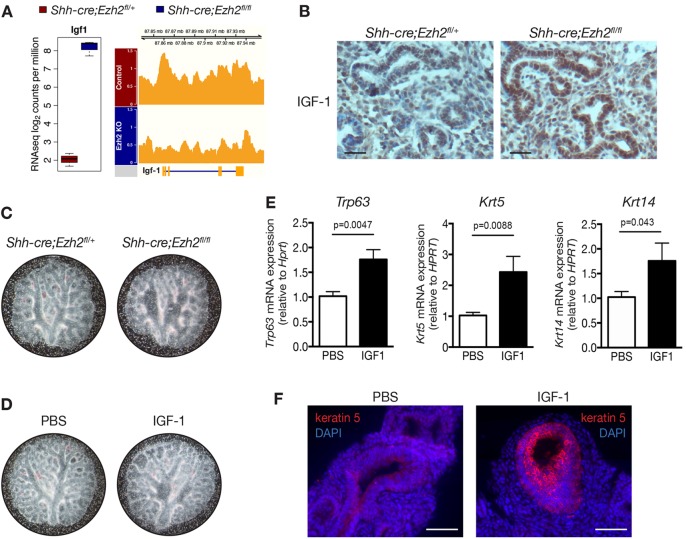
**IGF1 is upregulated in Ezh2-deficient lungs and promotes basal cell differentiation.** (A) Box plot showing RNA-seq expression values (normalized log_2_cpm, *n*=3, FDR<0.01) for *Igf1* and a genome browser view of H3K27 tri-methylation over the *Igf1* genomic locus. (B) Immunohistochemistry showing IGF1 expression in E15.5 lungs (representative of *n*=4). Scale bars: 50 µm. (C) Representative images of E11.5 control and *Shh-cre;Ezh2^fl/fl^* lungs cultured *ex vivo*. (D) Representative images of E11.5 wild-type lungs treated *ex vivo* with PBS or IGF1. (E) qRT-PCR analysis showing the expression of *Krt5*, *Trp63* and *Krt14* in PBS or IGF1-treated wild-type lungs. Results represent mean±s.e.m. *n*≥4 biological replicates. Unpaired *t*-test. (F) Immunofluorescence staining for keratin 5 in E11.5 wild-type lungs treated *ex vivo* with PBS or IGF1. Scale bars: 50 µm.

The transcription factor p63 is crucial for basal cell differentiation in the epidermis (Daniely et al., 2004) and overexpression of the transcriptionally active form of p63 (TAp63) ectopically in the lung has been shown to induce keratin 14 expression in distal airways (Koster et al., 2004). Surprisingly, some basal cell marker loci, including *Trp63* and *Krt5* did not appear to be marked by H3K27me3 (Fig. 5A; supplementary material Fig. S4B), suggesting that Ezh2 may indirectly regulate genes involved in basal cell differentiation. Overexpression of the Wnt antagonist Dickkopf homolog 1 (Dkk1) in the embryonic lung has previously been shown to induce basal cell differentiation in the distal lung (Volckaert et al., 2013). Our RNA-seq data showed that *Dkk1* was overexpressed in Ezh2-deficient epithelial cells (3.5-log_2_ fold increase, FDR<0.001) and that H3K27me3 marks were lost on the *Dkk1* promoter of Ezh2-deficient lungs (supplementary material Fig. S5A, FDR<0.001). However, *ex vivo* treatment of E11.5 *Shh-cre;Ezh2^fl/fl^* lungs with WAY262611, a specific inhibitor of Dkk1, did not inhibit the expression of *Krt5* or *Trp63*, suggesting that this pathway is not the only mediator of basal cell differentiation in Ezh2-deficient lungs (supplementary material Fig. S5B). Insulin-like growth factor 1 (*Igf1*) was also found to be highly upregulated in *Shh-cre;Ezh2^fl/fl^* epithelium compared with controls (Fig. 6A, 6.14-log_2_ fold increase, FDR<0.0001). ChIPseq analysis revealed that the *Igf1* locus was strongly marked with H3K27me3 marks in control animals but those marks were lost in *Shh-cre;Ezh2^fl/fl^* lungs (Fig. 6A, FDR=0.0003). Immunostaining for IGF1 showed a dramatic upregulation of IGF1 expression in the epithelium of Ezh2-depleted lungs compared with controls (Fig. 6B). IGF1 signaling has previously been implicated in the regulation of basal cell differentiation in the epidermis (Gunschmann et al., 2013), prompting us to investigate its role in mediating basal cell differentiation in the lung. When wild-type E11.5 lungs were treated *ex vivo* with IGF1, we observed dilatation of the airways, similar to what is observed in *Shh-cre;Ezh2^fl/fl^* lungs cultured *ex vivo* (Fig. 6C,D). Strikingly, increased expression of basal cell markers *Krt5* (2.4-fold), *Krt14* (1.7-fold) and *Trp63* (1.7-fold) was detected after treatment with IGF1 (Fig. 6E). Immunofluorescence staining further revealed the expression of keratin 5 in the upper airways of IGF1-treated lungs (Fig. 6F). FACS and western blot analysis confirmed an upregulation of keratin 5 and keratin 14 protein expression, as well as of Snai2, another marker of basal cell expression (Rock et al., 2009), after treatment with IGF1 (supplementary material Fig. S5C,D). However, treatment of E11.5 control or *Shh-cre;Ezh2^fl/fl^* lungs *ex vivo* with picropodophyllin (PPP), a specific inhibitor of IGF1R (Girnita et al., 2004) did not reduce *Krt5* or *Trp63* expression in Ezh2-depleted lungs (supplementary material Fig. S5B), indicating that inhibition of IGF1 signaling is not sufficient to prevent or reverse basal cell differentiation in the absence of Ezh2. In view of the dramatic genomic changes induced by loss of Ezh2 in the lung endoderm, a complex combination of factors is most likely involved in the regulation of basal cell differentiation after loss of Ezh2 in the endoderm. However, our results show that repression of *Igf1* expression by Ezh2 in lung epithelial cells is likely to be a crucial process for maintaining a proper control of lineage specification during embryonic lung morphogenesis.

## DISCUSSION

Epigenetic control of gene expression is essential for normal tissue morphogenesis. Here, we explore the role of the PRC2 histone methyltransferase Ezh2 in lung endoderm lineage specification. Ezh2-deficient lungs are abnormal and display impaired branching morphogenesis associated with perturbed epithelial lineage specification, lacking club cells but presenting Sox2-positive basal cells throughout the branching tree. We found that IGF1, a growth factor that is highly upregulated in Ezh2-depleted lung, induces basal cell differentiation *ex vivo* in wild-type lungs.

The expression of over 1600 genes is affected by Ezh2 loss in the lung endoderm, indicating that many factors are likely responsible for the lung phenotype of *Shh-cre;Ezh2^fl/fl^* animals. The expression of the cell cycle inhibitor genes *Cdkn2a/2b* is controlled by PRC2 and loss of Ezh2 in the epidermis leads to upregulation of *Cdkn2a/Cdkn2b* expression and reduced cell proliferation (Ezhkova et al., 2009). Similarly in the embryonic lung, we observed reduced proliferation of epithelial cells in *Shh-cre;Ezh2^fl/fl^* mice associated with an upregulation of *Cdkn2a/Cdkn2b* expression. Homeobox genes are known targets of PRC2 and are crucial for tissue patterning (Pearson et al., 2005). In the lung, Hox genes are predominantly expressed in the mesenchyme (Mollard and Dziadek, 1997) and *Hoxb5a* and *Hoxb5b* have been shown to be crucial for patterning of the airway lineages (Boucherat et al., 2013). The upregulation of mesenchymal Hox genes in the epithelium of *Shh-cre;Ezh2^fl/fl^* mice may be involved in the perturbed crosstalk between the endodermal and the mesenchymal compartments leading to altered myofibroblast differentiation; however, upregulation of Hox gene by treatment of wild-type E11.5 lungs with retinoic acid *ex vivo* was not enough to alter the phenotype of the lung, suggesting that other factors are likely to be involved. The mesenchymal niche plays a key role in the regulation of lung epithelial progenitor cell function where mesenchymal factors such as Wnt1 and Fgf10 promote epithelial differentiation (Hogan et al., 2014; Kumar et al., 2014; Volckaert et al., 2013). Further studies are required to explore the molecular changes that occur in the mesenchyme after loss of Ezh2 in the lung endoderm. Specific deletion of Ezh2 in the mesenchyme would also provide novel insights into its specific role in this compartment.

Lineage specification of the lung endoderm occurs through different waves of proliferation and differentiation. Sox9-positive progenitor cells drive the expansion of the branching tree before losing Sox9 expression and acquiring Sox2 expression (Alanis et al., 2014). Lineage-tracing experiments have shown that Sox9-positive or Id2-positive progenitor cells present at the tip of the epithelial tree before E15 behave as multipotent progenitor cells and drive branching morphogenesis (Alanis et al., 2014; Rawlins et al., 2009a). In *Shh-cre;Ezh2^fl/fl^* mice, initial branching of the lung primordia occurs normally with the formation of all pulmonary lobes. However, tertiary branching is affected by the loss of Ezh2, leading to reduction in size and number of branches as early as E14.5. This suggests that control of gene expression by Ezh2 is crucial for the regulation of progenitor cells present at the tip of the growing epithelial tree. Conditional deletion of Sox9 in the epithelium from E12 results in reduced lobe size and number of branches (Chang et al., 2013), a phenotype similar to Ezh2-conditional knockout mice, further implying that Ezh2 is required to control early multipotent progenitor cells.

From E16.5, perturbed airway lineage specification is observed in Ezh2-deficient epithelial cells with the absence of club cell differentiation and the appearance of basal cells, suggesting that Ezh2 also plays a role in the second wave of lung morphogenesis where differentiation of the conducting airway is driven by Sox2-positive progenitor cells. Sox2+ precursors generate airway lineage cells, including neuroendocrine, secretory and ciliated cells. In *Shh-cre;Ezh2^fl/fl^* mice, we observed an increase in ciliated cell numbers over secretory cells, similar to what has been described in mice where Notch signaling was abrogated in the lung epithelium (*Pofut^cnull^* and *Rbpjk^cnull^*) (Tsao et al., 2009). However, in contrast to our observation, Scgb3a2-expressing cells were also absent in the airways of *Pofut^cnull^* mice (Tsao et al., 2009). This suggests that Ezh2 controls terminal differentiation of secretory cells after Notch-mediated commitment towards secretory or ciliated cell lineages has occurred.

The most striking observation in *Shh-cre;Ezh2^fl/fl^* lungs is the proximalization of the distal airways with the presence of basal cells throughout the branching tree after loss of Ezh2 in the endoderm. These cells express Sox2, suggesting that they are derived from Sox2-positive progenitor cells. Ezh2 may act directly to prevent basal cell differentiation by depositing H3K27me3 repressive marks on basal gene loci but could also act indirectly by repressing genes that regulate basal cell differentiation. Indeed, although a large number of basal genes are enriched for H3K27me3 marks in the E16.5 lung endoderm, other basal cell-specific genes, such as *Trp63* and *Krt5* are not marked. We evaluated the role of Dkk1 and IGF1 in mediating basal cell differentiation after loss of Ezh2. These two factors have both previously been implicated in basal cell differentiation in the developing lung and in the epidermis, respectively (Gunschmann et al., 2013; Volckaert et al., 2013). Overexpression of Dkk1 in the embryonic lung leads to an increase in basal cell and secretory cell marker expression (Volckaert et al., 2013), in contrast to Ezh2-depleted lungs where basal cells are present exclusively surrounding the airways but secretory club cells are absent. Inhibition of Dkk1 activity *ex vivo* was not sufficient to inhibit the expression of basal cell-specific markers in Ezh2-depleted lung. Similarly, specific inhibition of IGF1 did not alter the expression of basal cell markers *ex vivo* in *Shh-cre;Ezh2^fl/fl^* lungs. However, *ex vivo* treatment of wild-type lungs with IGF1 induced basal cell marker expression in the upper airways. In the epidermis, loss of insulin/IGF1 signaling results in nuclear localization of FoxO proteins that trap p63 and prevent its binding to its target genes, inhibiting basal cell differentiation (Gunschmann et al., 2013). It is possible that a similar mechanism exists in *Shh-cre;Ezh2^fl/fl^* lung and that derepression of *Igf1* expression in the absence of Ezh2 induces basal cell differentiation by phosphorylating FoxO proteins, resulting in its cytoplasmic translocation, freeing up p63 that can then transactivate basal genes. Altogether, it is likely that, in the absence of Ezh2, de-repression of H3K27me3-marked basal genes, combined with perturbed IGF1 signaling, Dkk1 expression and other factors, are responsible for the proximalization of the distal airways. Snitow et al. recently described proximalization of the airways in *Shh-cre;Ezh2^fl/fl^* mice and suggested a role for *Pax9* in inducing basal cell differentiation (Snitow et al., 2015). *Igf1* was not identified in their gene expression analysis as a candidate gene upregulated after loss of Ezh2. Their microarray study was performed at E14.5 on whole embryonic lung, whereas our RNA-seq gene expression analysis was carried out at a later time point (E16.5) using sorted epithelial cells. This enabled us to enrich for epithelial-specific gene changes resulting in the identification of *Igf1*, among others, as a key target gene involved in basal cell differentiation.

In adult distal lung, basal cells are rare and expand in the context of flu-mediated lung injury, where they are thought to act as stem cells necessary for epithelial repair (Kumar et al., 2011; Rock et al., 2009; Zuo et al., 2015). It remains to be explored whether Ezh2 plays a role in the regulation of these progenitor cells in adult injured lung. Although a little controversial, Ezh2 appears important for fetal haematopoietic stem cell activity, whereas Ezh1, homolog of Ezh2, complements Ezh2 function to maintain haematopoietic stem cell (Mochizuki-Kashio et al., 2011; Xie et al., 2014). Similarly, Ezh1 and Ezh2 play redundant roles in hair follicle stem cells and a deletion of both is required to affect progenitor cell proliferation in the bulge (Ezhkova et al., 2011). It is therefore possible that, although Ezh2 is crucial for embryonic lung development, Ezh1 and Ezh2 can both regulate progenitor cell function in the adult lung. As distal basal cells are proposed to contribute to repair of the damaged lung, it is crucial to identify factors that regulate differentiation towards this lineage. Our observation that perturbed IGF1 signaling results in basal cell differentiation in the embryonic lung suggests that this pathway may also be important in the regulation of adult distal lung basal cells. Interestingly, immunostaining for IGF1 and its receptor in lung tissue from individuals with acute respiratory distress syndrome showed increased expression of IGF1 and IGF1R (Andonegui et al., 2014; Krein et al., 2003). It remains to be investigated whether increased secretion of IGF1 in adult injured lungs is crucial for basal cell expansion observed after epithelial cell damage.

### Conclusions

Our results demonstrate that Ezh2 is crucial for embryonic lung development and further reveal that Ezh2 tightly regulates epithelial cell lineage determination in the developing lung, consistent with its role in maintaining tissue specificity in other organs. Our data show that Ezh2 represses basal cell specification corroborating the recent findings by Snitow et al. (2015). We provide mechanistic insights into potential processes that mediate basal cell differentiation driven by the loss of Ezh2 in the developing lung epithelium. In particular, gene expression profiling and ChIP-seq studies enabled us to demonstrate that Ezh2 plays a novel role in maintaining epithelial cell lineage specification by depositing H3K27me3 repressive marks at the promoters of basal genes in the epithelium. We also show that repression of IGF1 signaling is an important mechanism for keeping basal cell specification genes transcriptionally silenced throughout lung development.

## MATERIALS AND METHODS

### Mouse strains

*Shh-Cre* mice (Harfe et al., 2004) were purchased from The Jackson Laboratory. *Ezh2^fl/fl^* mice were obtained from Prof. Tarakhovsky (The Rockefeller University, NY, USA) (Su et al., 2002) and Scgb1a1-creERT2 mice from Prof. Hogan (Duke University, NC, USA) (Rawlins et al., 2009b). All animal experiments were conducted according to the Walter and Eliza Hall Institute of Medical Research Animal Ethics Committee guidelines (AEC 2010.017).

### Histology and immunostaining

For histological examination, lungs were fixed in 4% paraformaldehyde in phosphate-buffered saline (PBS), embedded in paraffin, sectioned and stained with Haematoxylin and Eosin. For immunohistochemistry, sections were blocked in 10% serum prior to incubation with specific antibodies (see methods in the supplementary material) followed by a biotin-conjugated secondary antibody. For mouse primary antibodies, the Mouse on Mouse (M.O.M.) kit and the Biotin Blocking System were used according to the manufacturer's instructions (Vector). Signal was amplified using Vectastain Elite ABC Reagent (Vector) for 30 min followed by 3, 3′-diaminobenzidine (DAKO). Sections were counterstained with Haematoxylin. Quantification of Nkx2.1 and Foxj1 staining was automated through custom-written ImageJ Macros (using the FIJI distribution package). Segmentation was performed using the color deconvolution plug-in, combined with auto-threshold and size filtering. Cell quantifications were performed automatically, while diameter of the airway for Foxj1 quantification was manually defined.

For immunofluorescence staining, sections were blocked in 10% serum, incubated with appropriate antibodies overnight at 4°C followed by fluorophore-conjugated antibodies. Imaging was performed using a DeltaVision Elite microscope (Applied Precision). For Gomori's aldehyde fuchsin (GAF) staining, see methods in the supplementary material.

### RNA isolation and qPCR

Total RNA was extracted from embryonic lungs using either the Total RNA Purification Kit (Norgen) or miRNeasy Mini Kit (Qiagen). DNase treatment was performed on-column using RNAse-free DNAse I Kit (Norgen). cDNA was generated using SuperScript III (Invitrogen) from 500 ng of total RNA. qPCR was performed using the Sensimix SYBR Hi-Rox kit (Bioline) and primers described in the methods in the supplementary material. PCR was carried out in the Rotorgene RG-6000 and expression levels were normalized to *Hprt*. TaqMan probes were used for *Krt5*, *Krt14* and *Trp63* qPCR (see methods in the supplementary material) using Fast Advanced TaqMan mastermix.

### Fluorescence-activated cells sorting (FACS)

For FACS analysis of E18.5 lungs, individual lungs were digested in 500 μl of collagenase mix (1 mg collagenase/lung in DPBS+0.2 g glucose/liter) at 37°C for 30 min while shaking at 165 rpm, followed by red blood cell lysis with 0.64% ammonium chloride at 37°C for 3 min. Cells were resuspended in blocking solution (anti-FcR and Rat IgG) and incubated on ice for 10 min. Antibody staining was performed as described in the methods in the supplementary material. Cells were then washed and resuspended in PI solution. For cell cycle analysis, fixed and permeabilized cells (Fixe-Perm, BD) were stained with DAPI. Cells were analyzed using Fortessa1 and FortessaX20 or sorted using ARIA sorter (Beckton Dickinson). FACS data was analyzed using FlowJo9.6.2. Data represent mean±s.e.m. *P*-values were calculated using unpaired *t*-test assuming equal variance.

### *In vitro* proliferation assay

EpCAM^+^ sorted cells were plated on a 96-well low attachment plate in 100 μl media [DMEM:F12 with Glutamax, 100 U/ml penicillin, 100 μg/ml streptomycin, ITS, B27 (Gibco), 10 μg/ml EGF (Sigma), 20 μg/ml bFGF (R&D)]. After 3 days in culture, 10 μl of CellTiter 96 AQueous One Solution Reagent (Promega) was added to each well and incubated at 37°C for at least 1.5 h. Absorbance was measured at 490 nm.

### *Ex vivo* lung culture

E11.5 lungs were dissected and cultured at air-liquid interface on 8 µm membrane placed on 1 ml of media DMEM:F12 with Glutamax (Gibco) with 100 U/ml penicillin and100 μg/ml streptomycin. Lungs were treated for 3-4 days with DMSO, PBS, picropodophyllin (PPP) (150 nM, Santa Cruz Biotech), WAY262611 (500 nM, Millipore) or IGF1 (1 µg/ml, GroPep).

### Lung whole-mount staining and OPT imaging

Embryonic lungs were stained according to protocols described previously (Chang et al., 2013; Metzger et al., 2008). Briefly, E14.5 lungs and younger were fixed in 4% PFA at 4°C for 1 h while E15.5 and older lungs were fixed in DMSO:methanol (1:4) overnight at 4°C. Lungs were blocked in PBS with 5% serum and 0.5% Triton X-100, stained with keratin 5 or E-cadherin (see methods in the supplementary material) for 2-3 days and washed before adding the secondary antibody for 2 days. Lungs were imaged with optical projection tomography (OPT) to visualize the E-cadherin or keratin 5-positive structures of the lung. Lungs were quantified using two methods. Tree Surveyor software (Combes et al., 2014; Short et al., 2013) was used to quantify all aspects of accessory lobe branching from the E-cadherin OPT datasets (tertiary branches number, terminal sac number, lengths, volumes, diameters, curvature, angles). Imaris software (Bitplane, Oxford Instruments) was used to calculate whole lobe volumes using the Surfaces contouring tool. Statistical tests used were unpaired two-tailed *t*-tests for assessing lung and lobe volumes, and 2-tailed *t*-tests with Welch's correction for potential unequal variances for the accessory lobe statistical tests (terminal sac number, main branch number, airway length/volume).

### RNA-seq and ChIP-seq analysis

Reads were aligned to the mouse reference genome (*mm10*) using the Rsubread package (version 1.14.2) (Liao et al., 2013). For RNA-seq data, reads were summarized at the gene level using the featureCounts function in a strand-specific manner. The voom method (Law et al., 2014) was applied to transform the data and derive observational-level weights that were used in the fitting of gene-wise linear models (Ritchie et al., 2015) with TREAT (McCarthy and Smyth, 2009) to assess differential expression relative to a fold-change of 1.2. The ChIP-seq data was analyzed using the csaw package (Lun and Smyth, 2014) from the Bioconductor project to compare read depth in contiguous 2 kb bins between control and Ezh2-depleted lung epithelium. The data are available from GEO (Accession Numbers GSE57391 and GSE57392). For a detailed description of the analysis, refer to methods in the supplementary material. Gene set testing and microarray analyses are described in the supplementary material.

## Supplementary Material

Supplementary Material
