# Supplementary material for: Repression of Igf1 expression by Ezh2 prevents basal cell differentiation in the developing lung
Source: Development. 2015 Apr 15;142(8):1458–69. doi: 10.1242/dev.122077 (PMC4392602; doi:10.1242/dev.122077)
Supplement: Supplementary Material [file supp_142_8_1458__index.html]

Supplementary Material 

# Repression of *Igf1* expression by Ezh2 prevents basal cell differentiation in the developing lung

## DEV122077 Supplementary Material

**Files in this Data Supplement:**

- Supplementary Material
