# Supplementary material for: Repression of Igf1 expression by Ezh2 prevents basal cell differentiation in the developing lung
Source: Development. 2015 Apr 15;142(8):1458–69. doi: 10.1242/dev.122077 (PMC4392602; doi:10.1242/dev.122077)
Supplement: Supplementary Material [file supp_dev.122077_DEV122077supp.pdf]

Supplemental Figures

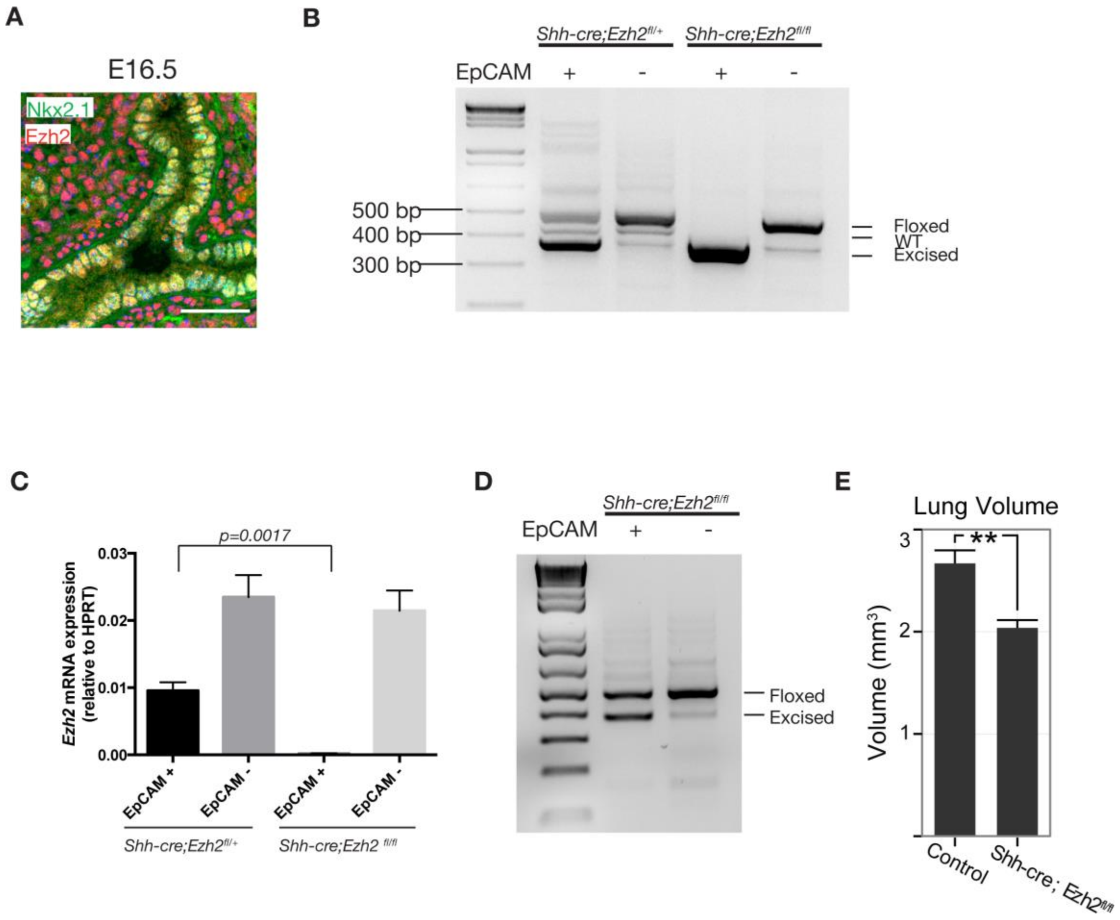

Figure S1. (**A**) Immunofluorescence staining of E16.5 lungs for Nkx2.1 (green) and Ezh2 (red) showing nuclear expression of Ezh2. Scale bars = 50µm. (**B**) PCR showing deletion of the SET domain of *Ezh2*. Genomic DNA was isolated from EpCAM^{+} and EpCAM^{-} sorted cells at E16.5. (**C**) RT-qPCR showing loss of the SET domain of *Ezh2* in the lung epithelium. (**D**) PCR showing incomplete excision of *Ezh2* SET domain in sorted EpCAM^{+} cells from one *Shh-cre;Ezh2$^{fl/fl}$* mouse that survived to adulthood. (**E**) Whole lung volume analysis of OPT scanned E14.5 *Shh-cre;Ezh2$^{fl/fl}$* and control lungs at E14.5. Data represent mean ± SEM. ** = p value < 0.01

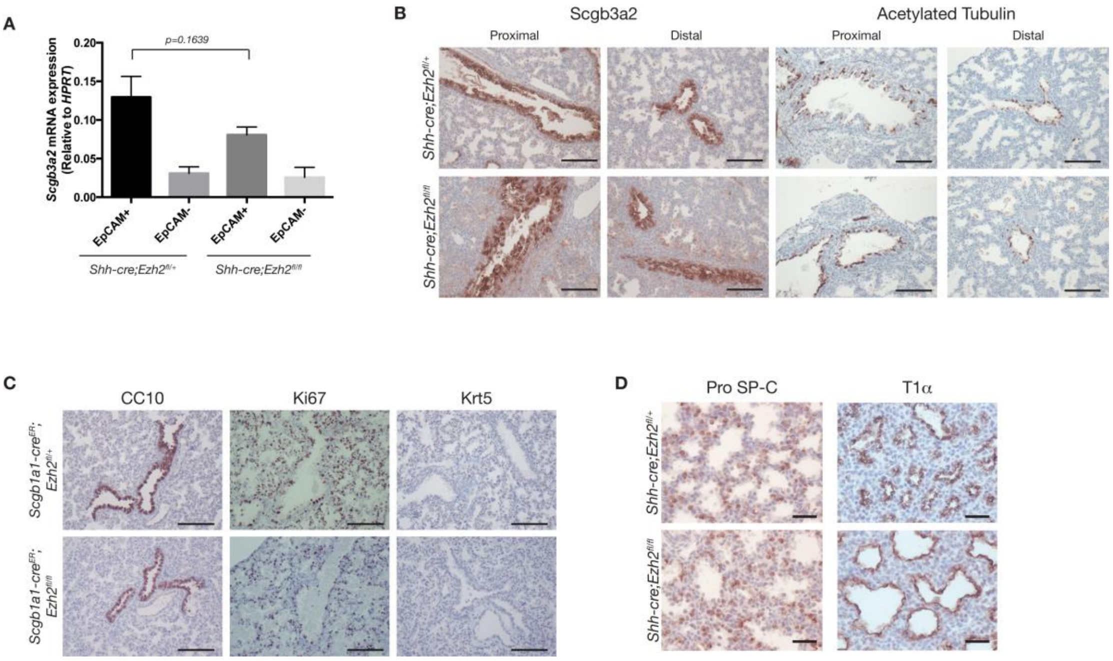

Figure S2. (**A**) RT-qPCR analysis of *Scgb3a2* mRNA expression in $EpCAM^+$ and $EpCAM^-$ cells isolated from E18.5 *Shh-cre;Ezh2*$^{fl/fl}$ and *Shh-cre;Ezh2*$^{fl/+}$ control mice. Data represent mean ± SEM (n=3). (**B**) Immunohistochemistry showing the expression of Scgb3a2 and acetylated tubulin in proximal and distal airways of E18.5 *Shh-cre;Ezh2*$^{fl/fl}$ and *Shh-cre;Ezh2*$^{fl/+}$ control mice (representative of n>7). Scale bars = 200 µm. (**C**) Immunohistochemistry staining for CC10, Ki67 and Keratin 5 in 1 week old *Scgb1a1-cre*^{ER}*;Ezh2*$^{fl/fl}$ and control animals. Scale bars = 200 µm. (**D**) Immunohistochemistry staining for markers of alveolar type II cell (Pro SP-C) in E18.5 lungs and alveolar type I cell (T1alpha) in E17.5 lungs. Scale bars = 25 µm.

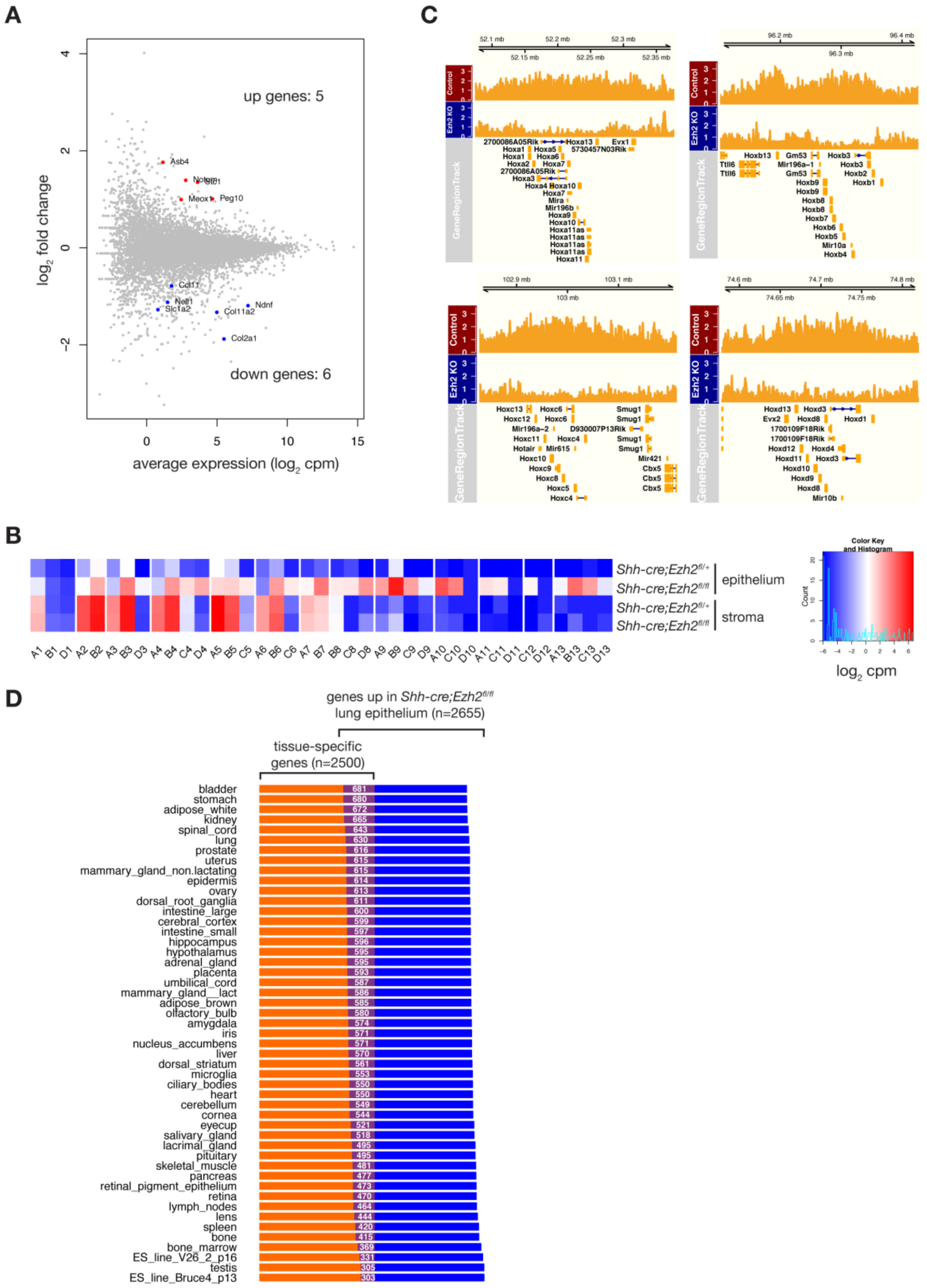

Figure S3. (**A**) MA plot showing differentially expressed genes between Ezh2-deficient and control stromal cells in E16.5 embryonic lung. Differentially expressed genes (FDR<0.05) are marked in red (up-regulated) and blue (down-regulated). (**B**)

Expression analysis of *Hox* genes in Ezh2-deficient and control lung epithelium and stroma. The colours in the heatmap represent average $\log_2$ cpm (n=3). (**C**) Genome browser view of H3K27 tri-methylation over the four *Hox* genomic loci demonstrating the loss of H3K27me3 mark in Ezh2-deficient epithelium. (**D**) Bar plot showing the number of overlapping genes (purple) between genes up-regulated in *Shh-cre;Ezh2$^{fl/fl}$* epithelium (blue) and top 2500 tissue-specific genes (orange).

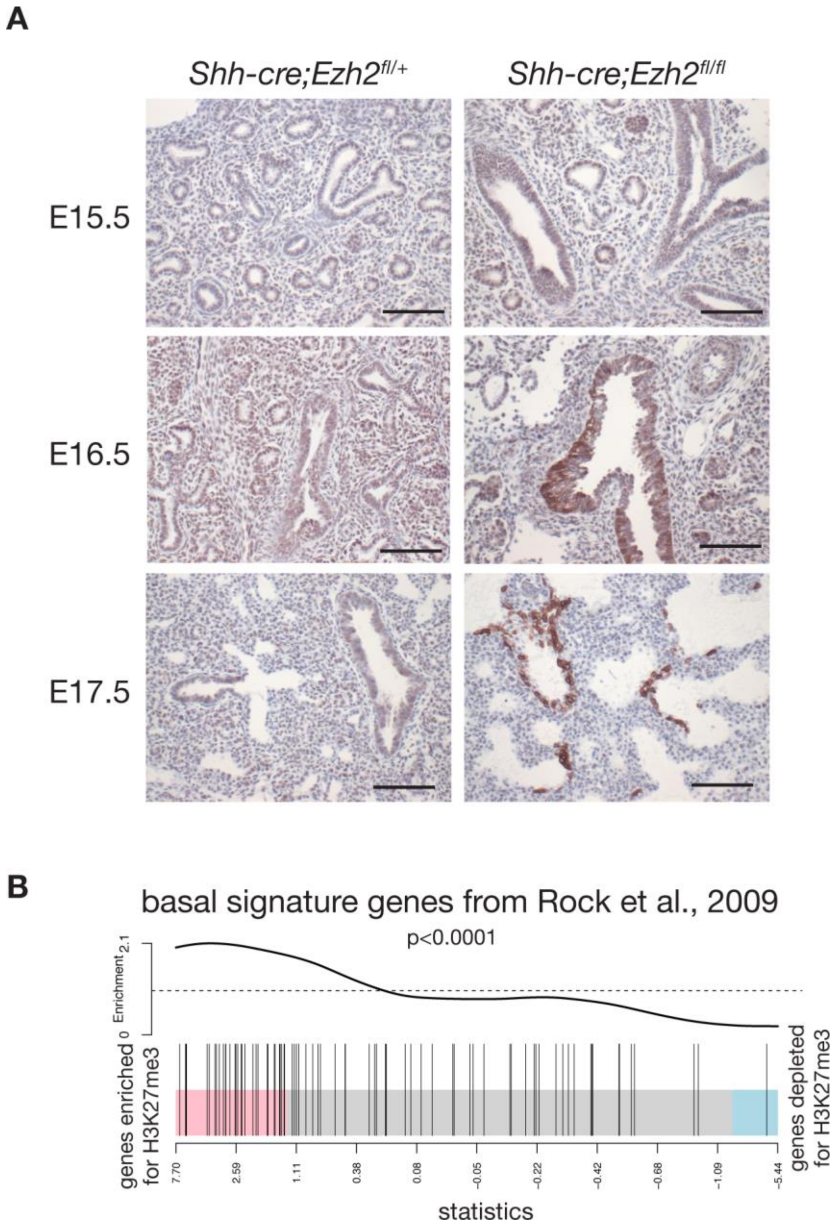

Figure S4. (**A**) Immunohistochemistry showing apparition of Keratin 5 expression from E16.5 in airways of *Shh-cre;Ezh2*$^{fl/fl}$ animals. Scale bars = 200µm (**B**) Gene set analysis of genes differentially marked by H3K27me3 (FDR < 0.05) in the basal gene signature from Rock et al. (2009) (gene set test p<0.0001).

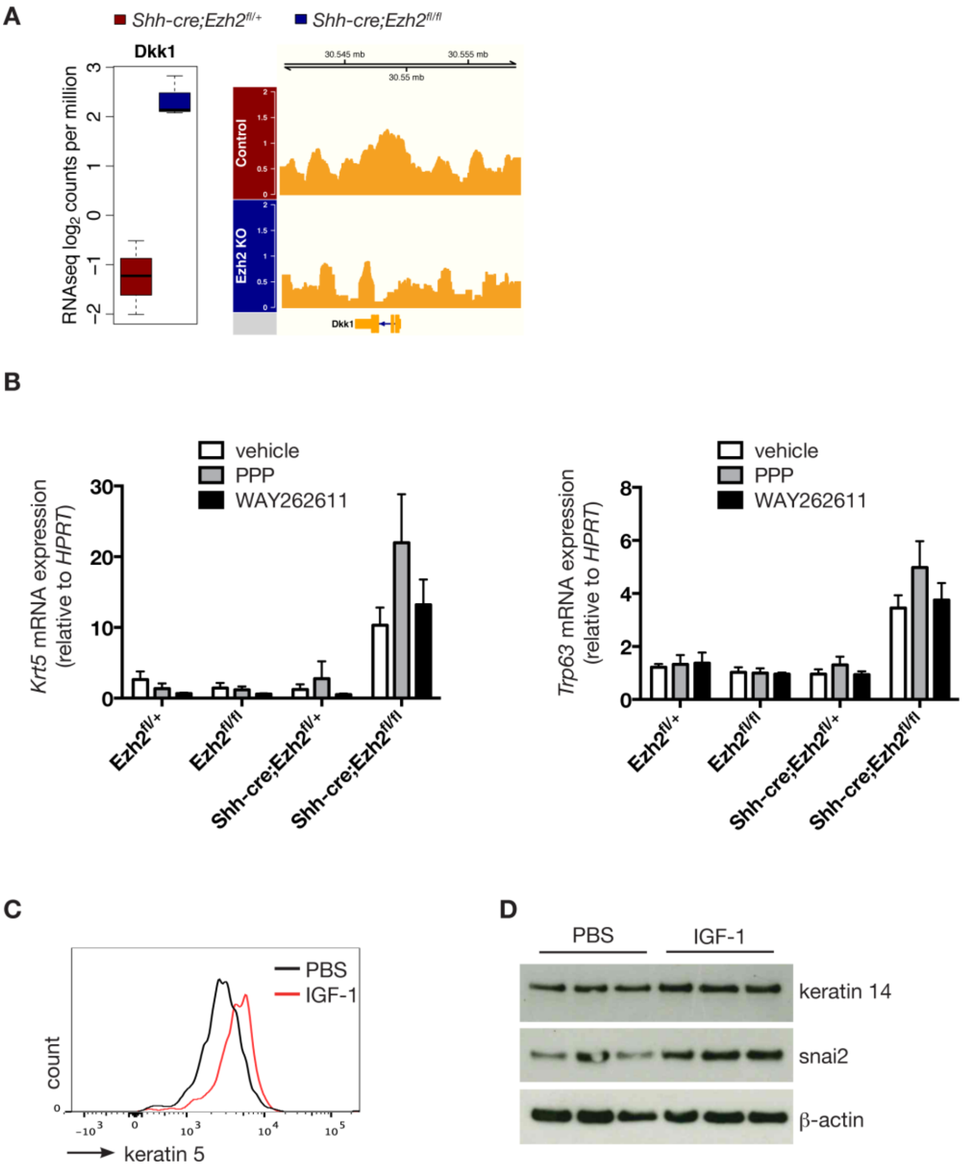

Figure S5. (**A**) Box plot showing RNA-seq expression values (normalised log_2 cpm, n=3, FDR<0.01) for *Dkk1* and genome browser view of H3K27 tri-methylation over the *Dkk1* genomic locus. (**B**) qPCR data showing the expression of *Krt5* and *Trp63* in control and *Shh-cre;Ezh2*$^{fl/fl}$ E11.5 lungs cultured *ex vivo* with WAY262611 (500nM) or PPP (150nM) for 72 hours. (**C**) Flow cytometry analysis of Keratin 5 expression in E11.5 wild-type lungs treated *ex vivo* with PBS or IGF-1 for 4 days. Cells gated are EpCAM$^+$CD104$^+$. (**D**) Western blot analysis showing the expression of Keratin 14, Snai2 and β-actin in E11.5 embryonic lungs cultured *ex vivo* with PBS or IGF-1 for 96 hours.

Movies

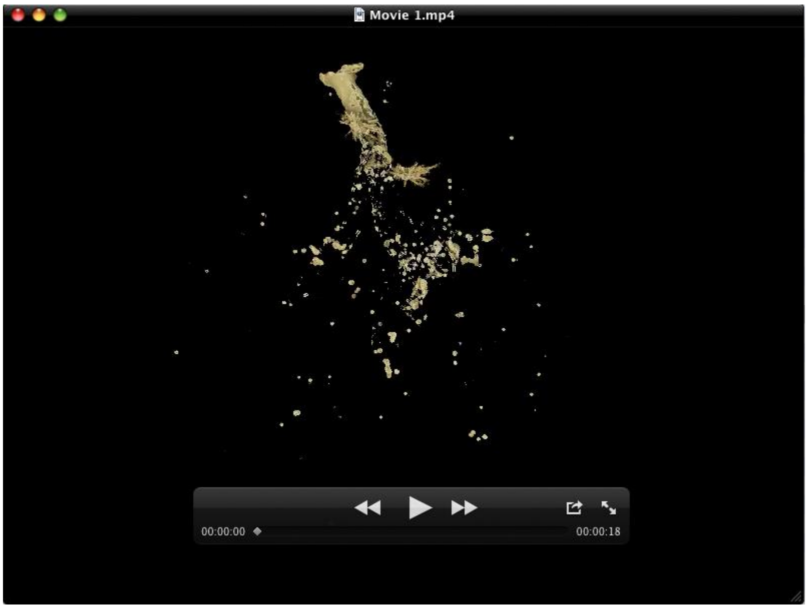

Movie 1: OPT-scanned three-dimensional imaging of *Shh-cre;Ezh2*$^{fl/+}$ E16.5 lung stained with Keratin 5.

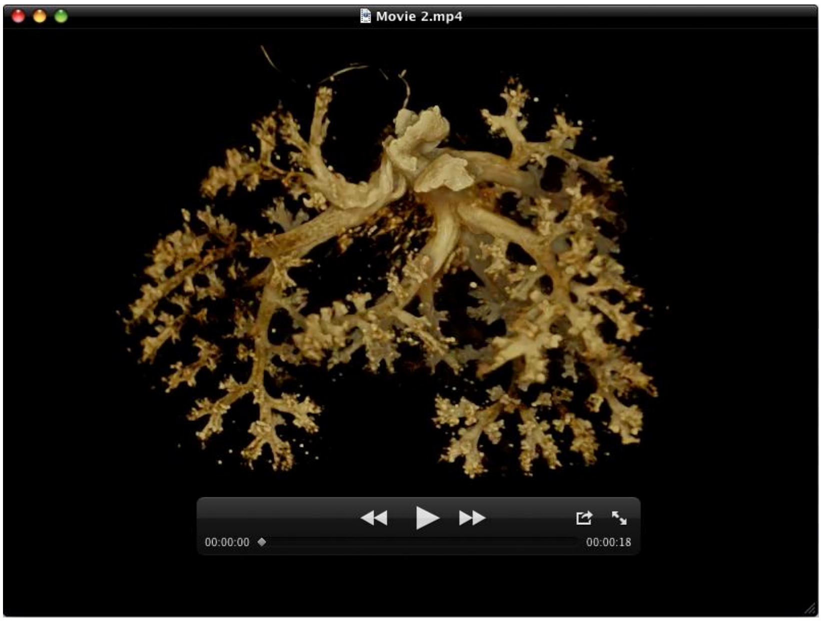

Movie 2: OPT-scanned three-dimensional imaging of *Shh-cre;Ezh2*$^{fl/fl}$ E16.5 lung stained with Keratin 5.

Table S1: Survival at weaning of offsprings from *Shh-cre* mice crossed with *Ezh2*$^{fl/fl}$ mice per genotype.

	Ezh2$^{fl/+}$	*Ezh2*$^{fl/fl}$	*Shh-cre; Ezh2*$^{fl/+}$	*Shh-cre; Ezh2*$^{fl/fl}$	Total
Observed # of offspring	56	36	43	1	136
Observed %	41.2%	26.5%	31.6%	0.7%	100%
Expected % of offspring	25%	25%	25%	25%	100%

X^2 test was used to compare expected and observed number of offspring. ($p= 1.46 \times 10^{-10}$)

Table S2

Click here to Download Table S2

Table S3

Click here to Download Table S3

Supplemental Methods

Table of primers used for genotyping and qPCR

**qRT-PCR Primers**	
Ezh2 SET Fwd	GAG GGC TAT CCA GAC TGG TG
Ezh2 SET Rev	ATG CCC ACA TAC TTC AGG GC
HPRT Fwd	CAC AGG ACT AGA ACA CCT GC
HPRT Rev	GCT GGT GAA AAG GAC CTC T
Scgb3a2 Fwd	GCT GGT ATC TAT CTT TCT GCT GGT G
Scgb3a2 Rev	ACA ACA GGG AGA CGG TTG ATG AGA
**Genotyping Primers**	
Ezh2 Fwd	TTA TTC ATA GAG CCA CCT GG
Ezh2 Fwd Left	ACG AAA CAG CTC CAG ATT CAG GG
Ezh2 Rev	CTG CTC TGA ATG GCA ACT CC
**Taqman probes**	
HPRT	Mm00446968_m1
Trp63	Mm00495793_m1
Krt14	Mm00516876_m1
Krt5	Mm00503549_m1

Table of Antibodies used for IHC, western blotting and FACS

**Antibody IHC/IF**	**Supplier**	**Catalogue number**	**Dilution**
CC10	Kind gift from Dr. B. Stripp (Regenerative Medicine Institute at Cedars -Sinai)	-	1/2000
Keratin 5	Covance	PRB-160P	1/1000
E-cadherin	Invitrogen	13-1900	1/500
FoxJ1	eBioscience	14-9965	1/400
Sox2	Millipore	AB5603	1/500
Sox9	Millipore	AB5535	1/4000
Nkx2.1	Dako	M3575	1/200
Acetylated Tubulin	Sigma-Aldrich	T7451	1/800
Ki67	Dako	M7249	1/200
Ezh2	Invitrogen	36-6300	1/250
Scgb3a2	R&D Systems	AF3465	1/100
T1alpha	DSHB	clone 8.1.1	1/1000
β-actin	Sigma	A5441	1/5000
IGF-1	Abcam	ab9572	1/100
Keratin 14	Covance	PRB-155P	1/1000

Snai2	Cell Signaling Technology	9585	1/1000
Pro SP-C	Seven Hills	WRAB-9337	1/1000

**Secondary Antibody**	**Company**	**Catalogue number**
Donkey anti- Rabbit	Invitrogen	A21207
Donkey anti-Rat	Invitrogen	A21208
Goat anti-IgG2a Mouse	Invitrogen	A21137
Donkey anti-Goat	Invitrogen	A21432
Donkey anti-Rabbit	Invitrogen	A21206
Goat anti-IgG1 Mouse	Invitrogen	A21240
Goat anti-Rabbit	Vector Labs	BA-1000
Rabbit anti-Rat	Vector Labs	BA-4001
Horse anti-Goat	Vector Labs	BA-9500

**Antibody FACS**	**Company**	**Dilution**	**Catalogue number**
EPCAM	Biolegend	1/200	118212/118218
CD31	Biolegend	1/250	102418
CD45	Biolegend	1/250	103114

Antibody staining for FACS analysis

Antibodies were diluted in 2%FCS/DPBS and incubated with cells for 25 min on ice. Cells were washed in 2%FCS/DPBS and incubated with secondary antibody for 15 min on ice. Cells were resuspended in Propidium Iodide solution before analysis on the flow cytometer.

GAF staining

Sections for Gomori's Aldehyde Fuchsin (GAF) staining were fixed on Bouin's fluid at 60°C for 1h, washed in water and left in 70% ethanol for 5 min. GAF stain was performed overnight at 4°C followed by 2h at RT. Sections were washed in 70% ethanol before Trichome staining and 1% acetic acid wash for 4 min each. Once washed in water, slides were incubated for 2 min in 2% light green in 1% acetic acid, washed in water and dehydrated for mounting.

RNA-seq: sample preparation and analysis

$EpCAM^+$ and $EpCAM^-$ cells from the Ezh2-deficient and control embryonic lungs at day E16.5 were pooled together as necessary to a minimum of 100000 cells per sample (3 samples per tissue-genotype combination). Total RNA was extracted and purified using Total RNA Purification Kit (Norgen) according to kit instructions. 150 ng of total RNA was subjected to NGS library preparation using TruSeq Stranded Total RNA with Ribo-Zero (Illumina) according to kit instructions. Completed libraries from different samples were pooled together at equimolar concentrations and sequenced on HiSeq 2000 with

TruSeq SBS Kit v3- HS reagents (Illumina) as 100 bp single end reads at the Australian Genome Research Facility (AGRF).

Reads were aligned to the mouse reference genome *mm10* and mapped to known genomic features at the gene level using the Rsubread package (version 1.14.2) (Liao et al. 2013) from the Bioconductor software project (Gentleman et al. 2004). Reads were summarized at the gene level using the featureCounts (Liao et al. 2014) function in a strand-specific manner. Genes with low counts were discarded, retaining only those with counts per million above 0.5 in at least 3 libraries. Predicted genes, genes without annotation and genes that mapped to Y or mitochondrial chromosomes were also removed from the analysis. After filtering, 14,831 genes remained available for the differential expression analysis. Read counts were normalised using the trimmed mean of M-values (TMM) method (Robinson and Oshlack 2010) from the edgeR package (version 3.8.2) (Robinson et al. 2010). The voom method (Law et al. 2014) was then applied to transform the data and derive observational-level weights which were used in the fitting of gene-wise linear models (Smyth 2004) with TREAT (McCarthy and Smyth 2009) to assess differential expression relative to a fold-change of 1.2. The false discovery rate (FDR) was controlled at 5% by applying the Benjamini-Hochberg method (Benjamini and Hochberg, 1995).

ChIP-seq: sample preparation and sequencing

Freshly sorted $EpCAM^+$ cells from Ezh2-deficient and control embryonic lungs at day E16.5 were cross-linked in 1ml of freshly prepared 1% formaldehyde for 10 min at room temperature (RT). Cross-linking was stopped by adding 100µl of 1.25M Glycine and 5

min incubation at RT. Cells were collected by centrifugation at 1000 x g for 5 min at 4°C, resuspended in 200µl of hypotonic buffer from Chromatrap Standard Pro-A ChIP spin column sonication kit (Chromatrap) containing 1µl of proteinase inhibitor cocktail (PIC) (Sigma) and incubated on ice for 10 min. At this stage concentration of nuclei in the hypotonic buffer was estimated using a haemocytometer. Nuclei were collected by centrifugation at 5000 x g for 5 min at 4°C. Nuclear pellet was snap-frozen in dry ice and stored at -80°C. Nuclear pellets from several preparations were pooled together to a minimum of 400000-500000 cells per sample (2 samples per genotype) in 130µl of cell lysis buffer (Chromatrap) containing 1µl of PIC and incubated on ice for at least 10 min. The entire volume of lysate was transferred to a microTUBE (Covaris) and sonicated on Covaris S220 machine for 15 min under the following conditions: Duty Cycle 2%; Peak Incident Power 105 W; Cycles per Burst 200.

Sonicated lysate was centrifuged at 10000 x g for 5 min at 4°C and resulting supernatant was collected and stored at -80°C. A 25µl aliquot of lysate was combined with 5µl of 1M $NaHCO_3$, 5 µl of 5M NaCl, 15µl dH20 and 1µl of 20mg/ml Proteinase K (Roche). The mix was incubated at 65°C for 2 hours and Proteinase K was inactivated by heating the mix to 95°C for 10 min. DNA was isolated by Phenol-Chloroform extraction followed by DNA precipitation and resuspended in 30µl of nuclease-free H_2O. The fragment size and DNA concentration were determined using D1K Screentape on Tapestation instrument (Agilent).

50µl of sonicated lysate was combined with 450µl Chromatin Dilution Buffer (Millipore), 10µg H3K27me3 antibody (Millipore #07-449), 2.25µl PIC (Sigma) and 40µl of Magna ChIP protein A magnetic beads (Millipore #16-661). The chromatin slurry was incubated overnight with rotation at 4°C and beads were washed according to Magna ChIP A kit instructions (Millipore #17-610). Chromatin was eluted from the beads by resuspension in 100µl elution buffer (1% SDS, 0.1M NaHCO3) containing 1µl of 20mg/ml Proteinase K (Roche) followed by incubation at 62°C for 2h with shaking and Proteinase K inactivation at 95°C for 10 min. 2 IP reactions were set up for each sample and eluted chromatin corresponding to the same sample was combined at this stage. DNA was isolated by phenol-chloroform extraction followed by precipitation and resuspended in 30 µl of nuclease free water.

20-30ng of immunoprecipitated DNA from each of the samples as well as 100ng of whole genome extract were subjected to NGS library preparation using TruSeq Nano DNA Sample Preparation Kit (Illumina) following kit instructions with the following adjustments: fragmentation and size selection steps were omitted and 10 cycles of amplification were carried out during the fragment enrichment step. Resulting libraries were size selected using Pippin Prep DNA Size Selection System (Sage Science) to ensure fragment size below 900 bp. Libraries were pooled at equimolar concentrations and sequenced on HiSeq 2500 TruSeq SBS Kit v3 - HS reagents (Illumina) as 100 bp single end reads at AGRF.

ChIP-seq analysis

Reads were aligned to the mouse reference genome mm10 using Rsubread package (version 1.13.25) (Liao et al. 2013). For visualisation purposes, genome browser tracks were plotted using Gviz package (version 1.7.10) (Hahne et al., 2013) with a 1000 bp smoothing window. Briefly, reads over genomic loci of interest were extracted from individual bam files and read coverage was normalised to the effective library sizes computed as described below. Read depth for control and Ezh2-deficient samples was further normalised by dividing read coverage at each position by the respective read depth of the whole genome extract sample.

We used a locally developed R software package (ChIP-seq analysis with windows, or csaw) based on a previously described approach (Lun and Smyth, 2014) to call H3K27me3 enriched regions. Reads from each library were counted into contiguous 2 kb bins spanning the entire genome. Reads in genomic regions annotated as repeat sequences according to RepeatMasker (Smit *et al.*, 2010) from the UCSC server (http://hgdownload.cse.ucsc.edu/goldenPath/mm10/bigZips/chromOut.tar.gz) were excluded from counting. The average log-count per million (logCPM) for each bin was computed using the aveLogCPM function in edgeR. Bins were filtered to retain only those with an average logCPM above 0.5 yielding 160484 bins. This removes low-abundance bins corresponding to putative regions of non-specific enrichment.

Normalization was then performed to correct for composition bias. Briefly, reads were counted in 10 kb bins for each library and the counts were used to compute normalization factors using the TMM method (Robinson et al. 2010). These factors were used to compute the effective library sizes for the differential enrichment analysis

performed using edgeR (Robinson and Oshlack 2010). Significant differences between control and Ezh2-deficient samples were detected for each 2 kb bin using the quasi-likelihood negative binomial framework (Lund et al. 2012).

For a promoter-based summary of H3K27me3 marking, the set of bins overlapping each promoter (defined as 3kb up- and downstream of transcription start sites (TSS)) was identified. A combined p-value was computed for each promoter using Simes' method. Promoters with significant differences in marking were detected after applying the Benjamini-Hochberg method on the combined p-values, to control the FDR across promoters at 5%. 1214 genes were identified with increased marking in the control samples over the knockouts. We also repeated the analysis aggregating bins over gene bodies (gene length plus 3kb upstream of TSS) with the similar outcome.

Gene set testing

Visualisation of gene set analyses was conducted using the barcodeplot function from the limma package (version 3.22.0) (Ritchie 2015). For each plot, the dataset of interest was ranked by moderated t-statistics and elements of queried gene sets or signatures were plotted as bars. Enrichment of the gene set elements across the range of the statistics was displayed by plotting a moving average calculated using a tri-cube weight function. Genes in the figure S4B were ranked by $\log_{10}$ of FDR signed by the direction of the fold change (i.e. genes enriched for the H3K27me3 mark in control samples were assigned positive value, while genes depleted for the H3K27 mark were assigned a negative value).

Focused gene set testing of lung basal cell signature (Rock *et al.*, 2009) was performed for differentially expressed genes between Ezh2-deficient and control lung

epithelium using the ROAST method (Wu et al. 2010). Gene set tests of H3K27me3 differentially marked genes was also performed among genes differentially expressed between conditions (Ezh2-deficient vs control lung epithelium) using ROAST and lung basal cell signature (Rock *et al.*, 2009) using the geneSetTest function from limma (Ritchie et al., 2015).

Microarray analysis

Tissue-specific expression analysis was carried out using publicly available GNF Mouse GeneAtlas V3 data (GEO accession number GSE10246). Samples corresponding to cell lines and blood cell types were removed leaving 49 solid tissues (including 2 ES cell lines). Expression values were $\log_2$ transformed and quantile normalised. Differential expression analysis was then carried out using linear modeling (Smyth 2004) by contrasting the average expression in each of the tissues to the average expression across the remaining tissues using the limma package (Ritchie et al., 2015). Gene-wise p-values were detected at a false discovery rate of 5% by applying the Benjamini-Hochberg method. This yielded between 2541 and 4625 differentially expressed genes per tissue (logFC > 0). We then defined tissue-specific signatures as the top 2500 differentially expressed genes (FDR < 0.05) ranked by logFC value and estimated the overlap between these tissue-specific signatures and the genes up-regulated in *Ezh2*-deficient epithelium (FDR < 0.05, logFC > 0, 2655 genes).

Basal cell expression signature was derived by re-analysing the expression microarray profile of mouse trachea basal cells (Rock et al., 2009, GEO accession number GSE15724). Probes without annotation and with non-unique gene IDs were

filtered out leaving 38557 probes. Differential expression analysis was carried out using the limma package with sample-specific weights (Ritchie et al., 2006) by contrasting gene expression in Krt5-GFP^{+};Lectin^{+} cells with the average expression of double negative (Krt5-GFP^{-};Lectin^{-}) and Krt5-GFP^{-};Lectin^{+} cells. To increase the stringency of the signature, we used the TREAT function in limma to test for differences greater than 2 fold. This approach yielded a basal signature of 165 unique genes that contained all of the consensus basal genes (Krt5, Krt14, Trp63, Ngfr, Snai2).

Supplemental References

Benjamini, Y., Hochberg, Y. (1995). Controlling the false discovery rate: a practical and powerful approach to multiple testing. Journal of the Royal Statistical Society Series B, 57, 289-300.

Gentleman RC, Carey VJ, Bates DM, Bolstad B, Dettling M, Dudoit S, Ellis B, Gautier L, Ge Y, Gentry J et al. 2004. Bioconductor: open software development for computational biology and bioinformatics. *Genome Biol* **5**.

Law CW, Chen Y, Shi W, Smyth GK. 2014. voom: Precision weights unlock linear model analysis tools for RNA-seq read counts. *Genome Biol* **15**: R29.

Liao Y, Smyth GK, Shi W. 2013. The Subread aligner: fast, accurate and scalable read mapping by seed-and-vote. *Nucleic acids research* **41**: e108.

Liao Y, Smyth GK, Shi W. 2014. featureCounts: an efficient general purpose program for assigning sequence reads to genomic features. *Bioinformatics* **30**: 923-930.

Lund SP, Nettleton D, McCarthy DJ, Smyth GK. 2012. Detecting differential expression in RNA-sequence data using quasi-likelihood with shrunken dispersion estimates. *Statistical applications in genetics and molecular biology* **11**.

McCarthy DJ, Smyth GK. 2009. Testing significance relative to a fold-change threshold is a TREAT. *Bioinformatics* **25**: 765-771.

Ritchie ME, Phipson B, Wu D, Hu Y, Law CW, Shi W, Smyth GK. 2015. limma powers differential expression analyses for RNA-sequencing and microarray studies. *Nucleic Acids Res*. [Epub ahead of print] PMID: 25605792

Ritchie ME, Diyagama D, Neilson J, van Laar R, Dobrovic A, Holloway A, Smyth GK. 2006. Empirical array quality weights in the analysis of microarray data, BMC *Bioinformatics* 2006, **7**:261

Robinson MD, McCarthy DJ, Smyth GK. 2010. edgeR: a Bioconductor package for differential expression analysis of digital gene expression data. *Bioinformatics* **26**: 139-140.

Robinson MD, Oshlack A. 2010. A scaling normalization method for differential expression analysis of RNA-seq data. *Genome Biol* **11**.

Smyth GK. 2004. Linear models and empirical bayes methods for assessing differential expression in microarray experiments. *Statistical applications in genetics and molecular biology* **3**: Article3.

Smyth GK. 2005. Limma: linear models for microarray data. in *Bioinformatics and Computational Biology Solutions Using {R} and Bioconductor*, pp. 397-420. Springer, New York.

Wu D, Lim E, Vaillant F, Asselin-Labat ML, Visvader JE, Smyth GK. 2010. ROAST: rotation gene set tests for complex microarray experiments. *Bioinformatics* **26**: 2176-2182.
